# Lysosomal Protease-Mediated APP Degradation is pH-Dependent, Mutation-Sensitive, and Facilitates Tau Proteolysis

**DOI:** 10.21203/rs.3.rs-6978813/v1

**Published:** 2025-07-10

**Authors:** Caroline Ackley, Zoe Liau, Shruti Arya, Tara Antee, Giselle M. Knudsen, Courtney Lane-Donovan, Paul J. Sampognaro, Aimee W. Kao

**Affiliations:** 1 Memory and Aging Center, Department of Neurology, University of California San Francisco, San Francisco, 100190, California, USA.; 2 Neuromuscular Division, Department of Neurology, University of California San Francisco, San Francisco, 100190, California, USA.; 3 Alaunus Biosciences, Inc., South San Francisco, 94080, California, USA.

**Keywords:** amyloid-precursor protein, APP, cathepsin, protease, amyloid-beta, A*β*, Alzheimer’s disease, lysosome, autophagy, tau, neurodegeneration

## Abstract

**Background:**

The accumulation and aggregation of amyloid beta (A*β*)—a peptide fragment derived from the proteolytic processing of amyloid precursor protein (APP)—is a central pathological feature of Alzheimer’s disease (AD) and a current target for disease-modifying therapies. Mutations in APP can also drive early-onset AD. While the roles of *α-, β-*, and *γ-*secretases and their respective cleavage sites in APP processing are well characterized, much less is understood about the routine degradation of APP within sub-cellular compartments like the lysosome.

**Methods:**

We applied Multiplexed Substrate Profiling by Mass Spectrometry (MSP-MS) to map cleavage sites within APP that may be targeted by lysosomal proteases, also known as cathepsins. We then employed cell-based and *in vitro* assays to examine the degradation of both wild-type and mutant APP by these enzymes.

**Results:**

Our findings confirm that APP is enriched in the endolysosomal compartment, where it is processed by many, but not all, cathepsins. Our experiments reveal that cleavages at several mapped APP sites are sensitive to both changes in pH and the presence of pathogenic variants E693G and E693Q. Additionally, we discovered that the large soluble domain of APP (sAPP) enhances tau cleavage by cathepsin G *in vitro*.

**Conclusions:**

Collectively, these results underscore the importance of lysosomal processing of APP, identify a link between APP and tau, and suggest new avenues for exploring AD pathogenesis. They also point to potential therapeutic targets related to the lysosomal function of APP and its impact on neurodegenerative disease.

## 1 Background

### 1.1 Proteolytic Processing of APP in Alzheimer’s Disease

Proteolytic processing of the amyloid-beta precursor protein (APP) is a central event in Alzheimer’s disease pathogenesis. Although extensive studies have characterized A*β* generation via secretases and caspases [1–6], the specific mechanisms by which APP processing contributes to neurodegeneration are not fully understood. To date, research has predominantly focused on a relatively narrow 37–49 amino acid segment of APP—the amyloid-beta (A*β*) peptide—even though full-length APP contains up to 770 amino acids (depending on the isoform) and full-length APP has been identified within amyloid plaques on autopsy [7]. Moreover, While cell surface APP is known to play crucial roles in neurodevelopment and synaptic plasticity [8], the functions and processing of the non-amyloid portions of APP within neurons remain largely unexplored. Additionally, while APP is known to accumulate and undergo cleavage into A*β* and other fragments within the endolysosomal system [9–17], a comprehensive characterization of its processing by lysosomal proteases is still lacking. Therefore, we set out to map the cleavage sites of these enzymes both to investigate novel mechanisms of A*β* release and also to shed light on the broader cellular processing of APP, potentially opening new avenues for understanding and also treating AD and related conditions.

### 1.2 Lysosomal Proteases and Neurodegeneration

A common hallmark of many neurodegenerative diseases, including AD, is the accumulation of misfolded proteins. Lysosomes, the “recycling centers” of cells, are critical for autophagy, protein homeostasis, and the clearance of protein aggregates resulting from such misfolded proteins. The degradative capacity of lysosomes is largely mediated by a diverse family of proteases known as cathepsins, which include serine, aspartyl, and cysteine proteases, all of which exhibit distinct substrate preferences [18]. Disruptions in lysosomal function—whether through alterations in pH [19–22] or genetic variants affecting lysosomal components [23, 24]—have been strongly implicated in neurodegeneration. Notably, an overabundance of APP fragments has also been shown to impede normal lysosomal activity [25–28]. Despite these associations, our understanding of how cathepsins process and degrade neurodegeneration-associated proteins remains incomplete. This gap is particularly critical given that impaired lysosomal proteolysis could exacerbate the accumulation of pathogenic proteins and thereby accelerate disease progression.

### 1.3 A Novel Approach to Mapping APP Cleavage

To address this critical gap, we employed Multiplexed Substrate Profiling by Mass Spectrometry (MSP-MS) on a custom-designed library of APP-derived peptides. By incubating these peptides with a panel of 13 lysosomal proteases—including 12 cathepsins and asparagine endopeptidase (AEP)—under varying pH conditions, our approach allowed us to delineate cleavage “hot spots” within APP as well as regions with low proteolytic susceptibility (e.g., the flexible acidic linker between the E1 and E2 domains). This comprehensive mapping not only revealed enzyme-specific cleavage patterns (with some sites being pH-dependent) but also provided predictive insights into how efficiently different proteases degrade soluble APP (sAPP). In particular, our data showed that proteolytic efficiency decreases with increasing pH, underscoring the importance of the lysosomal microenvironment in APP processing.

### 1.4 Linking APP Processing to Broader Neurodegenerative Mechanisms

Building upon our APP cleavage map, we compared these results with our previously published datasets on tau, TDP-43, and *α*-synuclein. Integrating these datasets enabled us to predict how pathogenic APP variants might alter lysosomal processing. Contrary to our initial hypothesis that deleterious variants would impede APP clearance, we observed that the Dutch (E693Q) and Arctic (E693G) variants actually enhanced proteolysis by lysosomal enzymes relative to the reference protein. This accelerated clearance was further corroborated in differentiated SH-SY5Y cells engineered to express mutant or wild-type APP. Moreover, we found that sAPP selectively promotes tau degradation *in vitro*, an unexpected discovery that hints at a previously unrecognized role for APP in modulating lysosomal proteolysis as well as the clearance of tau. Taken together, these findings build upon specific aspects of the Amyloid Cascade Hypothesis (ACH) and point towards novel therapeutic strategies targeting lysosomal processing pathways that may hold promise for modulating both APP and tau pathology in AD.

## 2 Methods

### 2.1 Recombinant proteases

Cathepsin A (CTSA) (R&D #1049-SE), Cathepsin B (CTSB) (Millipore #219,364), Cathepsin C (CTSC) (R&D #1071-CY), Cathepsin D (CTSD) (R&D #10140AS) for MSP-MS; (Sigma Aldrich #C8696) for all other assays, Cathepsin E (CTSE) (R&D #1294-AS), Cathepsin F (CTSF) (Abcam #ab240858), Cathepsin G (CTSG) (Millipore #219,873), Cathepsin H (CTSH) (R&D #7516-CY-010), Cathepsin K (CTSK) (Millipore #219,461), Cathepsin L (CTSL) (Millipore #219,402), Cathepsin O (CTSO) (Abcam #ab267932), Cathepsin S (CTSS) (R&D #1183-CY), Cathepsin V (CTSV) (R&D #1080-CY), Cathepsin X (CTSX) (R&D #934-CY), and asparagine endopeptidase (AEP) (R&D #2199-CY).

### 2.2 Antibodies

The following primary antibodies (commercial identifier, dilution) were used: 1) monoclonal rabbit anti-APP (Abcam #ab32126, 1:500), 2) monoclonal mouse anti-APP 22C11 (Invitrogen #14-9749-80, 1:1000, Figure 1C), 3) monoclonal mouse anti ATP5A1 (Invitrogen #459240, 1:500), 4) monoclonal rabbit anti-Cathepsin B (Abcam #ab214428, 1:1000), 5) monoclonal rabbit anti-Cathepsin D (Abcam #ab75852, 1:1000), 6) monoclonal mouse anti-EEA1 (BD #610457, 1:100), 7) monoclonal mouse anti-GAPDH (Abcam #ab8245, 1:1000), 8) polyclonal rabbit anti-GAPDH (Abcam #ab9485), 9) monoclonal mouse anti-HA (Invitrogen #26183, 1:500), 10) monoclonal mouse anti-Lamp1 (BioLegend #328611, 1:500, Figure 1A), 11) monoclonal rabbit anti-Lamp1 (Cell Signaling #9091S, 1:1000, Figure 1C), 13) monoclonal mouse anti-Lamp2 (DSHB #H4B4, 1:100), and 13) monoclonal mouse anti-tau (Santa Cruz Biotechnology, #sc-32274, 1:1000).

The following secondary antibodies were used for confocal microscopy: 1) polyclonal goat anti-rabbit AF488 (Thermo Scientific #A11008, 1:500, SH-SY5Y immunohistochemistry), 2) polyclonal goat anti-mouse AF647 (Life Tech #A21236, 1:500, SH-SY5Y immunohistochemistry), 3) polyclonal donkey anti-goat AF647 (Life Tech #A21447, 1:500, mouse cortex immunohistochemistry), 4) polyclonal donkey anti-mouse AF555 (Life Tech #A31570, 1:500, mouse cortex immunohistochemistry), and 5) polyclonal donkey anti-rabbit AF488 (Life Tech #A21206, 1:500, mouse cortex immunohistochemistry).

### 2.3 Generation of stable SH-SY5Y cell lines (APP WT, E693G, E693Q)

To generate stable APP-expressing SH-SY5Y lines, we first designed and synthesized a doxycycline-inducible, FLAG-tagged, full-length wild-type APP lentiviral construct using a puromycin-resistant plasmid backbone (pTet-O-Ngn2-Puro, Addgene #52047; Epoch Life Sciences). Starting from this wild-type construct, additional plasmids bearing single pathogenic point mutations (E693G and E693Q) were created. Lentivirus was produced using psPAX2 (Addgene #12260) and pCMV-VSV-G (Addgene #8454) packaging and envelope plasmids, respectively. SH-SY5Y cells already containing the pLenti CMV rtTA3 Blast construct (Addgene #26429) were infected and selected with 1 mg/mL puromycin. This resulted in three stable cell lines expressing either wild-type or mutant APP.

### 2.4 Animals

All mouse studies were conducted in accordance with the NIH guidelines and approved by the Institutional Animal Care and Use Committee (IACUC) of the University of California, San Francisco. To generate neuron-specific LysoTag mice, B6.129S4-Gt(ROSA)26Sortm1(CAG-TMEM192)Dmsa/J [29] mice were crossed with B6.Cg-Tg(Syn1-cre)671Jxm/J mice. These animals, maintained as heterozygotes, were used for all experiments. Mice were anesthetized with isoflurane for tissue collection. Perfusion with PBS followed by 4% paraformaldehyde was performed for immunohistochemistry, while fresh tissues for immunoprecipitation were collected after isoflurane sacrifice.

### 2.5 Immunohistochemistry (IHC)

Mouse cortex and cerebellum tissue was cryopreserved and sectioned into 30 *μ*m free-floating cortical slices using a cryostat. Sections underwent antigen retrieval in pH 8 Tris-EDTA at 80 °C for 30 minutes prior to blocking and antibody incubation.

Immunostaining of SH-SY5Y cells was performed in 8-well chamber slides (Ibidi #80841). Cells were examined either one day after plating (undifferentiated, Figure S1) or following a 10-day differentiation protocol with retinoic acid and BDNF [30]. They were then fixed in 4% paraformaldehyde for 15 minutes, washed with 0.1% Triton X-100 in PBS, and subsequently incubated with primary and secondary antibodies. Following antibody staining, all samples were sealed with Fluoroshield Mounting Medium with DAPI (Abcam #ab104139) and a coverslip.

#### 2.5.1 Microscopy

Following IHC, cells were imaged using a Nikon CSU-W1 spinning disk confocal microscope in the Nikon Imaging Center (NIC) at UCSF. Images were captured using a Plan Apo VC 100x/1.4 numerical aperture oil immersion lens and Andor Zyla sCMOS camera. DAPI, AF488, and AF647 fluorophores were stimulated using 405, 488, and 640 nm wavelength excitation, respectively.

#### 2.5.2 Colocalization analysis

Colocalization analysis of APP with organelle markers was performed using ImageJ/-FIJI software using a previously described method [31]. Briefly, a threshold was set for both APP and organelle marker channels to isolate the brightest 2% of pixels, and each remaining area was measured. The overlapping area was then calculated and divided by the thresholded APP area to determine the percentage APP colocalization with each marker (Figure S1).

### 2.6 Immunoprecipitation (IP)

LysoTag mice were sacrificed and fresh brain tissue collected for immediate IP as described previously [29]. Briefly, fresh brain tissue (cortex or cerebellum) was immediately homogenized in cold PBS using a glass dounce homogenizer (20 strokes). A portion of the homogenate was saved as a whole-cell control, while the remainder was mixed with 150 *μ*L of prewashed anti-HA magnetic beads (BioLegend #901501) and rotated at 4 °C for 20 minutes. Lysosomal fractions were separated using a magnetic rack, then resuspended in 1% NP-40 in PBS. After 10 minutes of rotation at 4 °C to remove beads, protein concentration was determined by BCA assay, and samples were stored at −80 °C for subsequent Western blot analysis.

### 2.7 Lysosome isolations

SH-SY5Y cells were differentiated as previously described [32, 33]. Cells were harvested 14 days post-differentation and their lysosomes were isolated using a gradient-ultracentrifuge based technique (Lysosome Enrichment Kit for Tissues and Cultured Cells from Thermofisher, #89,839). Successful isolation and enrichment of lysosomes were confirmed by Western Blot using a Lamp1 antibody.

### 2.8 Silver stains

Proteolytic assays were performed by incubating 1 *μ*g of recombinant human full-length APP/Protease Nexin II (R&D Systems #3466-PI) or a protease-specific control substrate with or without 1 *μ*M of individual proteases. Cathepsins A, C, H, and X were pre-activated according to the manufacturer’s instructions. Reactions were carried out for 1 hour at 37 °C in a total volume of 19.5 *μ*L using buffer conditions that included: 100 mM sodium citrate (pH 3.4), 50 mM sodium acetate (pH 4.5 or 5.5), or 100 mM PBS (pH 7.4), supplemented with 1 mM EDTA and 2 mM DTT. Protease activity was halted by adding 7.5 *μ*L of NuPAGE 4X LDS (Fisher Scientific #NP0007) and 3 *μ*L of 10X reducing agent (50 *μ*M) (Fisher #NP0009), followed by denaturation at 80 °C for 10 minutes. Samples were resolved using a NOVEX NuPAGE 4–12% Bis-Tris gel (Fisher #NP0321PK2) in MES buffer (Fisher #NP0002). After electrophoresis, the gel was fixed (40% ethanol, 10% acetic acid) and silver-stained according to the manufacturer’s instructions (Thermo Fisher #LC6070).

### 2.9 Multiplexed substrate profiling by mass spectrometry (MSP-MS)

MSP-MS was performed as previously described by [34] as well as our previous work [35]. First, a library of 18 amino acid-long peptides was created to cover the entire APP-770 sequence (Uniprot accession number P05067–1) with roughly 5 amino acid overlaps between fragments. To prevent aggregation resulting from disulfide bridges between peptides, all Cys residues were converted to Ala. Additional residues were added to both termini of each peptide to allow for liquid chromatography tandem mass spectrometric (LC-MS/MS) analysis. A complete table of peptides used in this library is provided in (Supplemental Table 1).

Lysosomal proteases were then incubated at 37 °C with the entire peptide library at 500 nM per peptide. Protease concentration ranged between 1–70 nM, with the exception of CTSF at 1 *μ*M. Reactions were monitored at 30, 60, and 240 minutes in an endpoint screening format. Aliquots were immediately desalted with C18 zip tips (Millipore-Sigma) and then freeze-dried. Samples were re-suspended in 0.1% formic acid in HPLC-grade water followed by LC-MS/MS analysis. For CTSA, which was pre-activated with CTSL, a matched CTSL-only negative control was compared for cleavage identification as described previously (Supplemental Table 2).

### 2.10 Mass spectrometry

Peptide sequencing by LC–MS/MS was performed on a QExactive Plus mass spectrometer (Thermo) equipped with a nanoACQUITY (Waters) ultraperformance LC (UPLC) system and an EASY-Spray ion source (Thermo). Reversed-phase chromatography was carried out with an EASY-Spray PepMap C18 column (Thermo, ES800; 3 *μ*m bead size, 75 *μ*m by 150 mm). Chromatography was performed at a 600-nl/min flow rate during sample loading for 14 min and then at a 400-nl/min flow rate for peptide separation over 90 min with a linear gradient of 2 to 35% (vol/vol) acetonitrile in 0.1% formic acid. Peptide fragmentation was performed by higher-energy collisional dissociation (HCD) on the six most intense precursor ions with a minimum of 2,000 counts, with an isolation width of 2.0 m/z and a minimum normalized collision energy of 25. Data were analyzed using Protein Prospector software, v.6.2.1 (http://prospector.ucsf.edu/prospector/mshome.htm, UCSF) using published methods [34]. The peptide cleavage data were then output as 8-mer sequences that spanned the P4–P4’ sites for each verified cleavage site (Supplemental Data).

### 2.11 *In silico* analysis of MSP-MS data

To identify specific cleavage sites following MSP-MS, enzyme-treated sample results were subtracted from a no-enzyme control incubation. Unique cleavage sites for each enzyme (i.e. all identified P1 sites for any pH condition or endpoint) were mapped for further analysis in R and through the generation of IceLogos. A Pearson correlation was performed to measure the degree of cleavage similarity between enzymes. The strength of each correlation with corresponding significance values were then plotted as a heatmap. A Euclidean distance matrix calculation followed by unsupervised hierarchical clustering was performed to compare cleavage patterns between APP and other proteins previously analyzed using MSP-MS [35].

### 2.12 Protease activity assays

Protease activity assays were performed in triplicate either in black, flat-bottom 384-well plates (Corning #3544) using custom designed fluorescent APP peptide substrates and FITC tau-441 (rPeptide #T-1113–1) or in Eppendorf Protein LoBind Tubes (Fisher Scientific #E925000090) using Recombinant Human APP (R&D Systems #3466PI010) and Recombinant Human 2N4R tau-441 (VWR #103790–836). Assays were conducted at 37 °C for six hours (fluorogenic assays) or 30 minutes (LoBind tube assays). The following buffers, each supplemented with 2 mM DTT and 1 mM EDTA, were used: 100 mM sodium citrate (pH 3.4), 50 mM sodium acetate (pH 4.5 or 5.5), or 50 mM phosphate-buffered saline (pH 7.4). In all reactions, final concentrations were as follows: 20 nM cathepsins, 20 *μ*M APP peptide substrates, 400 nM FITC-labeled tau-441, 5 *μ*M recombinant human tau-441, and 20–50 nM of APP or BSA.

#### 2.12.1 Fluorogenic APP peptide substrates

Each fluorescent APP peptide substrate consisted of an 8-residue sequence flanked by a 7-methoxycoumarin-4-acetic acid (MCA) fluorophore at the N-terminus and a dinitrophenol (DNP)-modified lysine quencher at the C-terminus (Supplemental Table 3). To enhance solubility, two arginine residues were included within each substrate. Before cleavage, the MCA signal is quenched by DNP. Proteolytic cleavage separates the fluorophore and quencher, resulting in detectable fluorescence. MCA fluorescence was measured using a BMG Labtech VANTAstar plate reader (excitation: 328 nm, emission: 393 nm).

#### 2.12.2 FITC tau-441

FITC tau-441 is a commercially available, full-length, recombinant, mature human tau isoform that is covalently labeled with Fluorescein Isothiocyanate (rPeptide #T-1113). Similar to the fluorogenic APP peptide substrates, it does not fluoresce when intact but becomes fluorescent when cleaved by protease activity. FITC fluorescence was monitored with a BMG Labtech VANTAstar plate reader using excitation and emission wavelengths of 485 nm and 530 nm respectively.

### 2.13 Western blots

For Western blot analysis, samples were denatured as described above and separated on pre-cast 4–12% NOVEX NuPAGE Bis-Tris gels using MOPS running buffer (Fisher Scientific #NP0001) and Invitrogen Novex Sharp Pre-Stained Protein Standards (Fisher Scientific #LC5800). Membranes were blocked at room temperature with Odyssey Blocking Buffer (LI-COR #927–40,100) and incubated at 4 °C overnight with primary antibodies and 1 hour at room temperature with fluorescent secondary antibodies (LI-COR, 1:5000). Immunoreactive bands were visualized using a LI-COR Odyssey CLx image scanner and quantified with Image Studio version 5.5.

### 2.14 Statistical analysis

Details of the statistical test used for each experiment is described in figure legends along with sample size and significance. All data is represented as mean ± standard error. Statistical analysis for plate reader assays was performed using GraphPad Prism 9 (GraphPad Software, La Jolla, California USA) while all other analysis was performed in R. Western blot quantifications for figures 4 and 5 were analyzed using 2-way, repeated measures ANOVA analyses. For all tests, a *p*-value <0.05 was considered significant.

#### 2.14.1 Heirarchical clustering analysis

Heirarchical clustering was performed to compare the relative contributions of each enzyme in cleaving APP (determined through MSP-MS) with our previously published analysis of tau, *α*-syn, and TDP-43 ([35]). The number of cleavages per enzyme was first divided by the total number of cleavage sites across each protein to calculate its contribution percentage. Because each protein differs in amino acid length, this percentage was then normalized to the mean contribution percentage of each enzyme across all four proteins. A Euclidean distance matrix comparing all normalized values was then calculated and an unsupervised single-link heirarchical clustering analysis was performed and visualized using R.

#### 2.14.2 Correlation analysis of cleavage profiles

To compare the cleavage profiles for each enzyme across APP, we performed a Spearman’s rank-order correlation analysis of the P1 sites identified through MSP-MS. Correlation coefficients and p-values were then plotted in a heatmap using R.

## 3 Results

### 3.1 APP is enriched in the endolysosomal compartment and is cleaved by lysosomal proteases *in vitro*

Although APP is classically described as a cell-surface transmembrane protein, recent evidence indicates that both APP and its cleavage products are also enriched in subcellular compartments—most notably within lysosomes [31, 36]. In addition to being endocytosed from the cell-surface, APP can be directly transported from the trans-Golgi network to endolysosomal compartments, bypassing the plasma membrane [12, 14]. Such trafficking is critical not only for the amyloidogenic processing of APP into A*β* [9, 10, 12, 14, 37] but also for the clearance of APP-derived byproducts [17, 25, 38].

**Figure 1.**
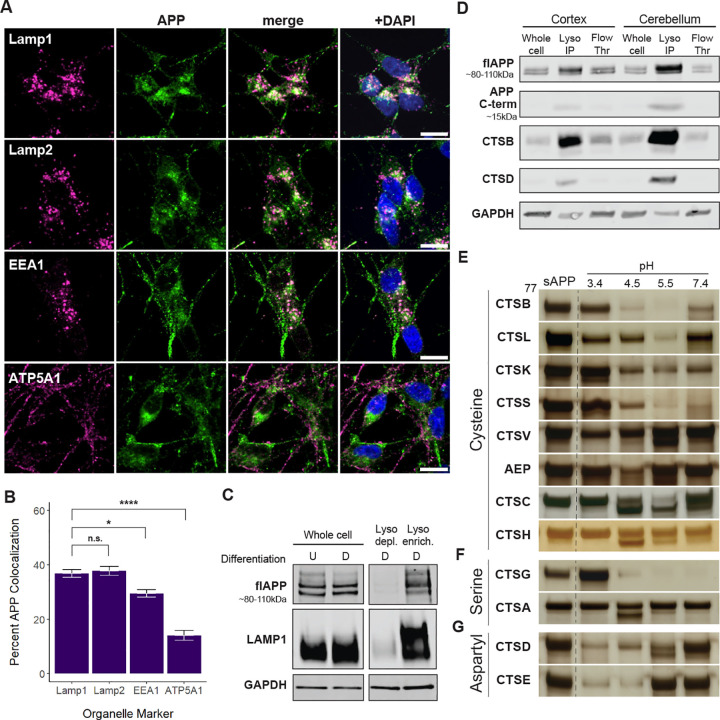
APP is enriched in the endolysosomal compartment and is cleaved by lysosomal proteases *in vitro*. **(A)** Differentiated SH-SY5Y neurons were fixed and immunostained with antibodies against organelle markers (magenta) and APP (green). Lamp1 and Lamp2, EEA1, and ATP5A1 are enriched in lysosomes, early endosomes, and mitochondria, respectively. Scale bar = 10 *μ*m. **(B)** Colocalization analysis was performed as described in methods. Percent APP overlap with each organelle marker was quantified and compared using a One-Way ANOVA followed by Bonferroni post-hoc analysis. * p <0.05, ** <0.01, *** <0.001. **(C)** Undifferentiated (“U”) and differentiated (“D”) SH-SY5Y cells were lysed and cellular contents were separated via density gradient centrifugation. Lysosome depleted (“Lyso depl.”) and lysosome enriched (“Lyso enrich.”) fractions were compared with whole cell samples on a Western Blot after staining with antibodies against APP, Lamp1, and GAPDH. **(D)** Cortex and cerebellum tissue was collected from LysoTag mice and neuronal lysosomes were collected via immunoprecipitation. Purified lysosomes (“Lyso IP”) were blotted alongside the non-immunoprecipitated flow through (“Flow Thr”) and whole cell samples. **(E)** Silver staining of sAPP cleaved by various lysosomal proteases and at multiple pH levels. Blots of enzymes that did not cleave sAPP are shown in Figure S3.

To further define APP’s intracellular distribution, we first examined its localization in SH-SY5Y neuroblastoma cells—a model relevant in both developmental and aging contexts. Using immunohistochemistry coupled with established colocalization analyses, we assessed APP overlap with lysosomal markers (Lamp1, Lamp2), an early endosomal marker (EEA1), and a mitochondrial marker (ATP5A1). Consistent with previous reports from other neuronal lines [31], APP colocalized with Lamp1 in differentiated (36.9%) and undifferentiated cells (44.45%), suggesting enrichment in the endolysosomal compartment (Figure 1A, Figure S1). In contrast, APP displayed far less colocalization with the mitochondrial marker ATP5A1 (14 and 17.4% for differentiated and undifferentiated cells, respectively). Western blot analyses of differentiated SH-SY5Y cells confirmed this lysosomal enrichment as well (Figure 1C).

Although APP was also detected at the plasma membrane and in other cellular regions in both undifferentiated (Figure S1) and differentiated cells (1), differentiated cells showed a distinct shift: APP localized to neuronal processes and exhibited reduced overall colocalization with multiple organelle markers, in line with its roles in synapse formation and cell adhesion (Figure 1A). Furthermore, a neuron-specific LysoTag mouse model—in which neurons express TMEM192-HA for lysosome immunoprecipitation [29] (Figure S2)—further demonstrated that full-length APP, in addition to the lysosomal proteases cathepsin B (CTSB) and cathepsin D (CTSD), is enriched in lysosomal fractions from both cortex and cerebellum compared with whole-cell lysates (Figure 1D).

Given that much of APP is lysosomally located, we next assessed which cathepsins were capable of cleaving the lysosomal lumen-facing portion of APP, also known as soluble APP (sAPP). While previous studies have shown that cathepsins B, D, L, and X can cleave APP and/or A*β* [38–42], there has never been a systematic assessment of cathepsins that cleave sAPP. To address this gap, we incubated recombinant soluble APP (sAPP) with individual cathepsin proteases at pH 3.4, 4.5, 5.5, and 7.4: conditions that span the broad potential activity range of these enzymes. In doing so, we found that most cysteine proteases efficiently cleaved sAPP (Figure 1E, Figure S3–S4). In general, these cysteine proteases preferred higher pH values (4.5–7.4). Interestingly, CTSH exhibited cleavage against APP, which was surprising given our prior work demonstrating a relative lack of activity against *α*-synuclein, tau, or TDP-43 under similar conditions [35]. Notably, CTSV—which we previously found to preferentially cleave *α*-synuclein, tau, and TDP-43 at pH 3.4 [35]—exhibited a much broader activity profile for sAPP, showing cleavage at pHs 3.4, 4.5, and 5.5.

Among the serine proteases, CTSG stood out by displaying robust activity across pH 4.5, 5.5, and 7.4. CTSA also demonstrated cleavage of APP at pH 4.5, while not previously displaying any significant activity against *α*-synuclein, tau, or TDP-43 in this experimental paradigm [35]. Lastly, the aspartyl proteases, CTSD and CTSE, both cleaved sAPP and favored more acidic pHs. Similar to CTSV, both CTSD and CTSE exhibited a broader range of activity on sAPP than tau, TDP-43, or *α*-synuclein.

Together, these findings reveal a diverse pH-dependent landscape for cathepsin-mediated APP processing. They highlight the unique ability of APP among neurodegenerative disease-relevant substrates to be cleaved by CTSA and CTSH, and underscore the importance of systematically profiling these enzymes to better understand their potential roles in Alzheimer’s disease.

### 3.2 MSP-MS provides a detailed cleavage map of APP by lysosomal proteases

To obtain a comprehensive view of lysosome-dependent APP turnover, we next employed MSP-MS to map cleavage sites across the full-length APP sequence. Although select cathepsin cleavage sites within the A*β* region have been identified [39, 40], the full spectrum of lysosomal protease activity on APP has not been previously characterized.

**Figure 2.**
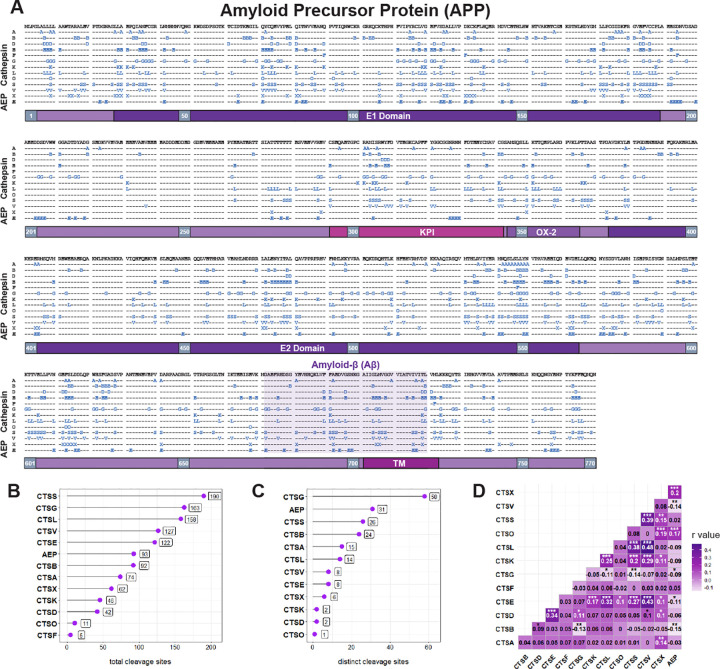
MSP-MS provides a detailed cleavage map of APP by lysosomal proteases. **(A)** A comprehensive cleavage map of APP generated through MSP-MS. P1 cleavage sites are indicated with a correpsonding letter (i.e. Cathepsin A = “A”), or by the symbol “Æ” for Asparagine Endopeptidase (AEP/legumain). **(B)** A bar graph representing the number of unique cleavage sites across the sequence of APP-770 for each enzyme. **(C)** Distinct cleavage sites—defined as P1 sites that were only identified for a single cathepsin—were quantified and are displayed as a bar graph. **(D)** A correlation plot visualizing the similarity in cleavage sites between enzymes. Darker values represent a greater degree of overlap between proteases. * p <0.05, ** <0.01, *** <0.001.

To perform these experiments, we generated a custom library of overlapping 18-amino acid peptides spanning the longest human APP isoform (APP-770) and incubated this library with 13 different proteases under various pH conditions and time points (Supplemental Table 1, Figure S5). This approach revealed a total of 390 unique cleavage sites, with each enzyme generating between 5 (CTSF) and 190 (CTSS) sites (Figure 2A–B). All proteolytic cleavage sites are shown, although presumably, only the amino acids in the sAPP region (roughly positions 1–700) would be oriented towards the lysosomal lumen. With these experiments, we confirmed that many of the cathepsins previously shown to efficiently cleave recombinant sAPP, such as CTSS, CTSG, and CTSL, yielded the highest number of cleavage events. In contrast, CTSF and CTSO produced fewer events overall. The total cleavage sites for each protease are shown in Figure 2B.

Interestingly, while CTSL and CTSS were prominent contributors to APP cleavage (paralleling their roles in processing other neurodegeneration-related substrates), APP was generally less biased toward any one protease compared with tau, *α*-synuclein, or TDP-43 (Figure S6). We also observed that cleavage events were unevenly distributed along the APP sequence; the E1, KPI, and E2 domains contained many highly redundant protease cleavage sites, whereas the flexible linker between E1 and the KPI was notably resistant to cleavage (Figure 2A).

Analysis of “hot spots” and “quiet zones” also revealed differences in cleavage redundancy. Some sites were targeted by multiple proteases, while others were uniquely cleaved by a single enzyme. We also observed that CTSG in particular represented a large proportion of these “distinct” cleavage sites (58 of 195 total sites, or approximately 13.8%), which was nearly twice the number of sites targeted by the next enzyme, AEP (Figure 2C). Overall, roughly half of all identified sites (195 of 390) were cleaved by a single protease, suggesting that even small shifts in protease activity or expression during disease could substantially impact APP processing.

To assess further the redundant versus non-redundant activity among these proteases, we also performed pairwise correlation analyses. We found that CTSS and CTSL, which cleave at 48.7% and 40.5% of identified cleavage sites, respectively, also showed considerable overlap with CTSE, CTSK, and CTSL. As expected, the cleavage profiles of the aspartyl proteases cathepsins D and E were positively correlated with one another (Figure 2D). Meanwhile, enzymes with distinctive cleavage sites, such as AEP, CTSS, and CTSA, exhibited limited overlap with others. Much like our previous study of *α*-syn, tau, and TDP-43, CTSB exhibited weak or negative correlations with most other cathepsins, further indicating that its loss of function might disproportionately impair neuronal lysosomes.

### 3.3 Lysosomal proteases exhibit unique substrate specificities that are pH-sensitive

Lysosomes typically maintain an acidic pH (approximately 4.0–4.5), which is optimal for many proteases to recognize and cleave specific substrates. While it is well-known that altered protonation states of proteases may impair substrate binding (thereby reducing cleavage efficiency), we wondered how changes in pH might affect the lysosomal proteases specifically and whether their substrate specificity—the ability of a protease to selectively target particular peptide bonds—might potentially be sensitive to pH-induced changes as well.

**Figure 3.**
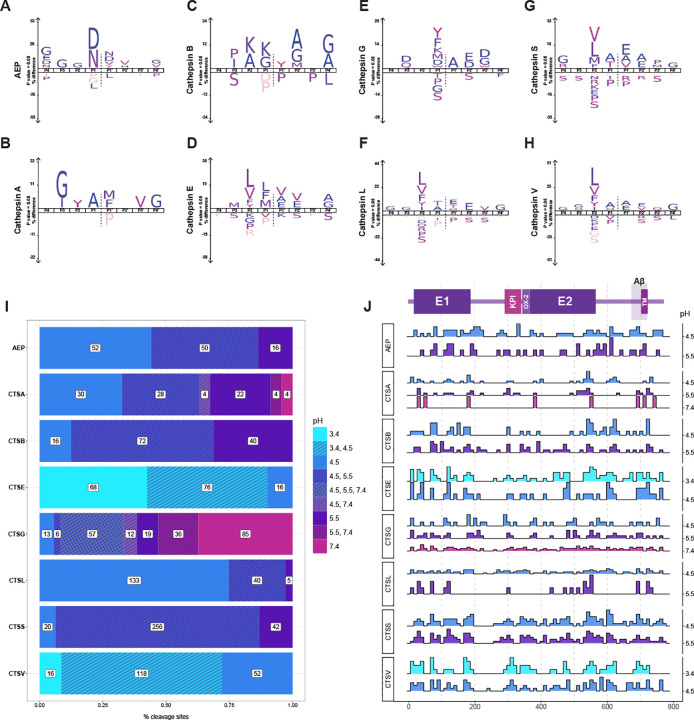
Lysosomal proteases exhibit unique substrate specificities that are pH-sensitive. **(A–H)** IceLogos generated for select enzymes based on MSP-MS data (additional IceLogos available in Figures S6–S7). **(I)** Stacked barplots representing the number of cleavage sites tallied at each pH **(J)** Graphical overview of the APP-770 protein and a map of cleavage patterns across APP at varying pH levels. Dotted lines occur every 100 amino acids. E1, E2 = E1 and E2 domains, respectively. KPI = Kunitz Protease Inhibitor domain. TM = transmembrane. Histograms were generated at each experimental pH value and protease combination tested using MSP-MS. Bar heights indicate the relative number of cleavage sites for each condition (ie. the area under each plot = 100%).

To this end, we first created IceLogos for each enzyme to assess their individual substrate specificity and compared these profiles with known motifs from the MEROPS database [43] (Figure 3A–H, Figure S7–8). Most cysteine proteases (cathepsins B, L, S, V, K, and AEP) proteases and the aspartyl proteases (cathepsins D and E) displayed cleavage patterns consistent with established substrate preferences. However, the substrate preferences for the serine proteases (cathepsins A and G) are less well-described. As such, we integrated our APP dataset with our previous work on tau, *α*-synuclein, and TDP-43 [35], to shed light on the substrate specificities of these enzymes (Figure S9). In doing so, we found that CTSA had a substrate preference for non-polar residues between P3-P3’, whereas CTSG exhibited a preference for large chained amino acids or those with aromatic rings.

Lysosomal pH is known to become more alkaline with age in yeast and *C. elegans* [44, 45]. To assess how age-associated ph changes can affect APP processing, we compared pH-dependence of cleavage for each protease (Figure 3I–J). We found that most proteases cleaved most efficiently at their known pH optima as referenced in MEROPS and the Brenda enzyme database [43, 46]. However, we were surprised to find that a one-unit increase in pH dramatically altered the activity of most of these enzymes. For example, a change in pH from 4.5 to 5.5 resulted in an impressive ~84% reduction in CTSL activity. Given CTSL’s broad neuronal expression and central role in our cleavage map, this dramatic pH sensitivity may help explain diminished APP and fragment clearance in less acidic lysosomes—a phenomenon observed in disease contexts [20, 41, 47]. Furthermore, many of these pH-dependent changes were observed within the A*β* region, underscoring their potential relevance to AD pathology.

### 3.4 Disease-associated mutations alter cathepsin cleavage of A*β* region

Extensive research has focused on secretase-mediated cleavage of APP and has carefully characterized the pathogenic mechanisms leading to A*β* accumulation in neurodegeneration [48, 49]. For example, the AD-causing Swedish mutation (KM670/671NL) enhances *β*-secretase processing, leading to increased A*β* production, while the London mutation (V717I) shifts *γ*-secretase activity to favor the more amyloidogenic A*β*42 isoform [50, 51]. By contrast, the A673T variant in APP reduces *β*-secretase cleavage and lowers amyloid accumulation, thereby decreasing AD risk and acting as a “protective” variant [52]. While many of these mutations highlight the importance of secretase cleavage in AD, there are other pathogenic variants in APP, such as the Arctic (E693G) and Dutch (E693Q) variants, that are removed from secretase cleavage sites (i.e., in the middle of the A*β* region) and whose pathogenic mechanisms are less well understood.

**Figure 4.**
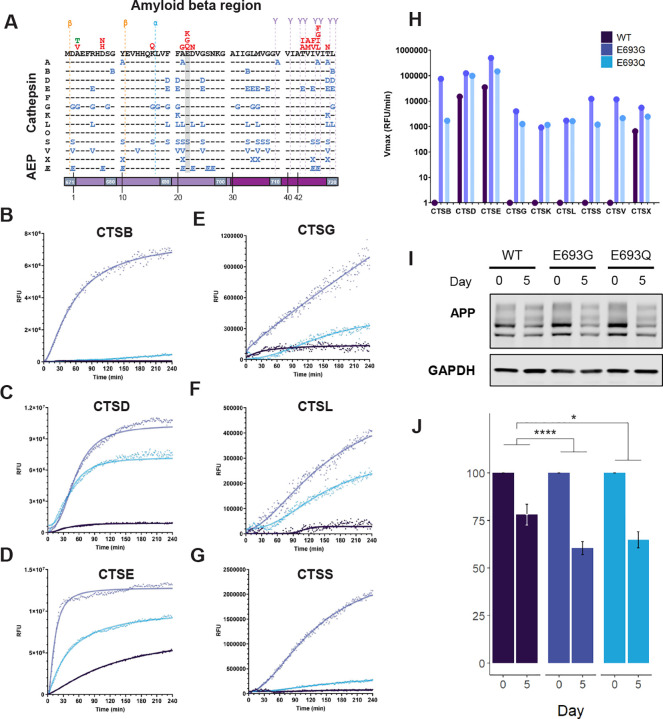
Disease-associated mutations alter cathepsin cleavage of A*β* region. **(A)** Expanded A*β* region of the MSP-MS cleavage map from Figure 2. Dotted lines represent known *β*, *α*, and *γ* cleavage sites. Protective (green) and deleterious (red) variants are plotted above the primary sequence. A*β* numbering is shown in black. Site E693 is highlighted with a grey bar. **(B–G)** E693 variants alter cleavage by lysosomal proteases. Wild-type, Arctic (E693G), and Dutch (E693Q) fluorogenic peptides were incubated with various cathepsins. Cleavage activity was determined by quantifying fluorescence over time. **(H)** Maximum reaction velocity (Vmax) of the cleavage assays shown in **(B–G)**. RFU/min are plotted on a logarithmic scale. **(I)** Differentiated wild-type and E693 variant SH-SY5Y cells were collected at 0 and 5 days following doxycycline induction of APP expression. **(J)** Band intensity from **(I)** was quantified and normalized to GAPDH on Western Blots (8 replicates). * p <0.05, ** <0.01, *** <0.001.

The pathogenic Arctic (E693G) and Dutch (E693Q) variants are associated with AD and cerebral amyloid angiopathy, respectively (Figure 4A). In contrast to other A*β* mutations, previous experiments have demonstrated that mutations at position 693 are associated with reduced A*β* levels, an increase in A*β* N-terminal fragments, and little to no amyloid PiB uptake in human studies [53–55]. While prior studies have suggested that these observations may be due to more rapid oligomerization and fibrillization of these variant fragments compared to wild-type [56–59], our newly generated APP cleavage map proposes an alternative hypothesis: with so many lysosomal protease cleavages present at position 693, we hypothesized that altered lysosomal processing of APP could contribute to diminished levels of A*β*.

To test this hypothesis, we employed fluorescence-based protease activity assays using short peptide substrates labeled with a fluorophore and quencher. These substrates, spanning the E693 region, were synthesized with either the wild-type sequence or the E693G/Q mutations. Upon incubation with 20 nM of cathepsins, both the E693G and E693Q peptides were cleaved more rapidly than the wild-type peptide (Figure 4B–G). In particular, CTSD, CTSL, and CTSG exhibited significantly enhanced activity toward the mutant peptides across a range of optimal pH conditions. For example, in the CTSL assays, the E693G/Q peptides were readily cleaved, while the wild-type substrate showed minimal activity. Furthermore, no protease displayed a reduction in activity with the mutant substrates compared to wild type (Figure S10).

To validate these findings in a cellular context, we generated stable SH-SY5Y lines harboring an inducible, FLAG-tagged APP that was either wild type or mutant at position 693. SH-SY5Y cells can be terminally differentiated into neuron-like cells. After differentiation, they were collected at day 0 and day 5 post-doxycycline induction and APP levels were quantified by Western blot. Over five days, APP levels in cells expressing either the E693G or E693Q variant decreased by ~40% on average, compared with an ~25% decrease in wild-type cells (Figure 4H–I, Figure S11).

### 3.5 APP augments Tau cleavage by Cathepsin G

While it is well-known that amyloid plaques precede tau aggregation and pathology, often by many years or even decades, a direct causative link between amyloid aggregation and the development of tau accumulation remains elusive. Prior studies have examined the potential toxic gain of function of amyloid oligomers and aggregates as well as potential loss of function mechanisms [60–62]. In examining the interplay of APP with the lysosomal proteases, we took particular note of the role of APP (also known as Protease Nexin-II) as a potent serine protease inhibitor of enzymes such as thrombin and trypsin [63]. We therefore hypothesize that APP might serve as an inhibitor of the serine lysosomal proteases, potentially altering tau degradation.

**Figure 5:**
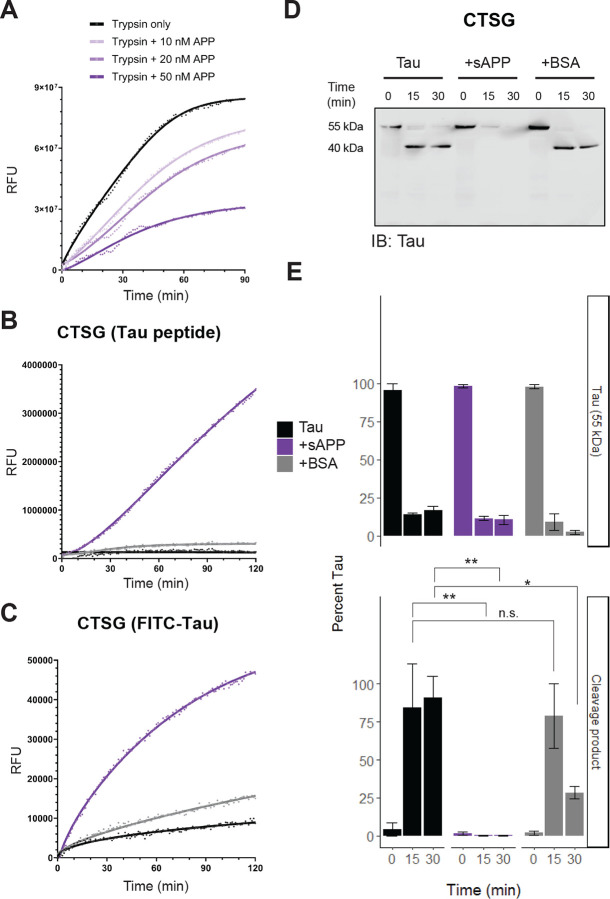
APP augments Tau cleavage by Cathepsin G. **(A)** Activity assay measuring cleavage of a fluorogenic APP peptide in the presence of increasing Trypsin concentrations. **(B–C)** Activity assays measuring the cleavage of a fluorogenic tau-peptide **(B)** and FITC-labelled tau **(C)** by Cathepsin G over time in the presence of either sAPP or BSA. **(D)** Recombinant human tau-441 was cleaved *in vitro* by Cathepsin G in the presence of either sAPP or BSA and analyzed via Western Blot. **(E)** Quantification of **(D)**, measuring tau (55 kDa) as well as a prominent cleavage product (40 kDa) (3 replicates). * p <0.05, ** <0.01, *** <0.001.

To begin, we confirmed that sAPP does indeed inhibit trypsin in a dose-dependent manner (Figure 5A). Notably, sAPP did not alter CTSG cleavage of the same casein-based, “universal” substrate (Figure S12). To our surprise, however, CTSG cleavage of tau increased rapidly in the presence of sAPP (Figure 5B–C). We observed this with both a tau-specific peptide fragment as well as full-length FITC-labeled tau as substrates. This augmentation of CTSG activity led us to question whether APP might serve a neuroprotective role by facilitating the lysosomal clearance of tau, positing that APP loss of function may subsequently lead to reduced tau clearance over time.

Extending these observations further, we also measured tau degradation in the presence or absence of sAPP by incubating recombinant, full-length, unlabeled tau 441 with CTSG at pH 4.5. Because we noticed a partial benefit from the addition of bovine serum albumin (BSA) in our activity assays (likely due to the effects of molecular crowding), an equimolar concentration of BSA was also included. Under these conditions at pH 4.5, nearly complete tau clearance was observed after 15 minutes when sAPP was present (Figure 5C–D, Figure S13). By contrast, CTSG alone or with BSA produced only partial tau cleavage, leading to the emergence of a prominent 40 kDa fragment within this same timeframe.

## 4 Discussion

This study provides a comprehensive characterization of lysosomal protease-mediated degradation of APP, revealing novel insights into the pH-dependence, mutation-sensitivity, and broader implications of this process for AD. Our findings extend existing models of APP processing, highlighting the lysosome as a critical, yet underappreciated, site of APP metabolism and a potential therapeutic target.

We first confirmed that APP is significantly enriched within the endolysosomal system of both neuronal cell lines and mouse brain tissue, consistent with previous reports [12, 13, 17, 64]. This localization is crucial, as it places APP in direct contact with the lysosomal proteases, primarily cathepsins, responsible for its degradation. Our *in vitro* assays using recombinant sAPP demonstrated that a wide range of cathepsins, including cysteine (CTSB, CTSC, CTSH, CTSK, CTSL, CTSS, CTSV, CTSX), serine (CTSA, CTSG), and aspartyl (CTSD, CTSE) proteases, can cleave APP. This broad susceptibility contrasts with the more selective cleavage patterns we previously observed for other neurodegeneration-related proteins like tau, *α*-synuclein, and TDP-43 [35]. The finding that CTSH, CTSV, and CTSA cleave APP, but not the other tested substrates, highlights a unique aspect of APP’s lysosomal processing.

Next, our MSP-MS analysis provides, to our knowledge, the first comprehensive map of lysosomal protease cleavage sites across the full-length APP-770 isoform. This map reveals a complex landscape of proteolytic susceptibility, with “hot spots” of high cleavage activity in the E1, KPI, and E2 domains, and a relatively resistant flexible linker region. The identification of both redundant cleavage sites (targeted by multiple proteases) and distinct sites (specific to a single protease) suggests that shifts in lysosomal protease expression or activity, which can occur during aging and disease [18, 23, 65], could significantly alter APP processing and potentially contribute to AD pathogenesis.

Critically, our study demonstrates the pH-dependence of cathepsin-mediated APP cleavage. Most proteases exhibited activity profiles consistent with their known pH optima, but we observed a particularly striking sensitivity for certain cathepsins, such as CTSL, with activity drastically reduced by even small pH increases. Given the established role of lysosomal dysfunction in AD and other neurodegenerative diseases [20, 47, 66, 67], this finding suggests that even subtle changes in lysosomal pH could significantly alter or impair APP clearance. Such a phenomenon could potentially occur with aging, physiological stress or neurodegenerative disease, potentially contributing to the accumulation of APP fragments and exacerbating disease progression.

Our investigation into the Arctic (E693G) and Dutch (E693Q) APP mutations revealed a surprising and potentially significant finding: these mutations enhanced cleavage by several key lysosomal proteases, including CTSD, CTSL, and CTSG and suggests that A*β* has a more complicated relationship with protease activity than was previously appreciated.

Perhaps our most surprising finding is the dramatic enhancement of tau proteolysis by CTSG in the presence of sAPP. This observation unveils a previously unrecognized interaction between APP and tau within the lysosomal environment. Although sAPP (also known as Protease Nexin-II) is a well-established serine protease inhibitor, its *augmentation* of CTSG’s activity on tau, rather than inhibition, is counterintuitive. This suggests a complex underlying mechanism, potentially involving allosteric modulation of CTSG, altered substrate presentation, or the formation of a ternary complex comprising sAPP, tau, and CTSG. Such a facilitating role within the lysosome is not without precedent; for instance, progranulin has been shown to facilitate the maturation of CTSD, thereby enhancing its proteolytic activity [68]. Similarly, saposin C is known to enhance the activity of glucocerebrosidase on glucosylceramide [69]. Further investigations are currently underway to elucidate the precise molecular mechanism of this newly described lysosomal function of sAPP.

This finding carries significant implications for understanding the intricate interplay between APP and tau pathologies in AD. It raises the compelling possibility that APP, beyond its role as the source of A*β*, may directly influence tau clearance within the lysosome. Consequently, a loss of this sAPP-mediated enhancement of tau degradation could directly contribute to the accumulation of pathological tau, thus offering a novel perspective on AD progression.

Acknowledging the inherent challenges in fully replicating physiological complexity, this study does possess certain limitations. While our *in vitro* assays offer valuable, detailed insights into protease activity, they cannot entirely recapitulate the intricate environment of a living cell. Future research will therefore extend to more compex cellular bodels, including primary neurons and induced pluripotent stem cell (iPSC)-derived neurons from patients carrying APP variants, to validate these findings in a more biologically relevant context. Furthermore, while our LysoTag mouse model served as a valuable tool for investigating lysosomal APP processing *in vivo*, additional studies are necessary to confirm these observations in human brain tissue, an area that has seen limited extensive past exploration [70]. Finally, the exact molecular mechanism by which sAPP enhances CTSG-mediated tau degradation warrants comprehensive investigation.

## 5 Conclusion

Our study provides compelling evidence that lysosomal protease-mediated degradation of APP is a complex, pH-regulated, and mutation-sensitive process with significant implications for AD pathogenesis. The detailed cleavage map generated by MSP-MS, the unexpected effects of the Arctic and Dutch mutations, and the novel discovery of sAPP’s role in augmenting tau degradation by CTSG, all highlight the importance of considering the lysosome as a central hub in the interplay between APP and tau pathologies. These findings open new avenues for research and suggest that therapeutic strategies targeting lysosomal function, specifically modulating cathepsin activity or enhancing sAPP’s interaction with tau and CTSG, may hold promise for treating AD and related neurodegenerative diseases.

## Supplementary Files

This is a list of supplementary files associated with this preprint. Click to download.

- SupplementaldataAckleyetal.csv
- SupplementalTablesAckleyetal.pdf
- SupplementalFiguresAckleyetal.pdf

**Supplementary information.** If your article has accompanying supplementary file/s please state so here.

Authors reporting data from electrophoretic gels and blots should supply the full unprocessed scans for key as part of their Supplementary information. This may be requested by the editorial team/s if it is missing.

Please refer to Journal-level guidance for any specific requirements.

## Data Availability

All data generated or analyzed during this study are either included in this published article (Supplemental Data) or are available from the corresponding author on reasonable request. An interactive protein visualization tool is available online for APP MSP-MS data at https://apps.rtemis.org/rtemisseq/.
